# Deep-Breathing Biofeedback Trainability in a Virtual-Reality Action Game: A Single-Case Design Study With Police Trainers

**DOI:** 10.3389/fpsyg.2022.806163

**Published:** 2022-02-10

**Authors:** Abele Michela, Jacobien M. van Peer, Jan C. Brammer, Anique Nies, Marieke M. J. W. van Rooij, Robert Oostenveld, Wendy Dorrestijn, Annika S. Smit, Karin Roelofs, Floris Klumpers, Isabela Granic

**Affiliations:** ^1^Behavioral Science Institute, Radboud University, Nijmegen, Netherlands; ^2^Faculty of Behavioral, Management and Social Sciences, University of Twente, Twente, Netherlands; ^3^Donders Institute for Brain, Cognition and Behavior, Radboud University, Nijmegen, Netherlands; ^4^NatMEG, Department of Clinical Neuroscience, Karolinska Institutet, Stockholm, Sweden; ^5^Faculty of Law, Radboud University, Nijmegen, Netherlands; ^6^Police Academy of the Netherlands, Apeldoorn, Netherlands; ^7^Humanism and Social Resilience, University of Humanistic Studies, Utrecht, Netherlands; ^8^Faculty of Social Sciences, McMaster University, Hamilton, ON, Canada

**Keywords:** police education, police training, virtual-reality, stress management, autonomic arousal, heart rate variability, biofeedback

## Abstract

It is widely recognized that police performance may be hindered by psychophysiological state changes during acute stress. To address the need for awareness and control of these physiological changes, police academies in many countries have implemented Heart-Rate Variability (HRV) biofeedback training. Despite these trainings now being widely delivered in classroom setups, they typically lack the arousing action context needed for successful transfer to the operational field, where officers must apply learned skills, particularly when stress levels rise. The study presented here aimed to address this gap by training physiological control skills in an arousing decision-making context. We developed a Virtual-Reality (VR) breathing-based biofeedback training in which police officers perform deep and slow diaphragmatic breathing in an engaging game-like action context. This VR game consisted of a selective shoot/don’t shoot game designed to assess response inhibition, an impaired capacity in high arousal situations. Biofeedback was provided based on adherence to a slow breathing pace: the slower and deeper the breathing, the less constrained peripheral vision became, facilitating accurate responses to the in-game demands. A total of nine male police trainers completed 10 sessions over a 4-week period as part of a single-case experimental ABAB study-design (i.e., alternating sessions with and without biofeedback). Results showed that eight out of nine participants showed improved breathing control in action, with a positive effect on breathing-induced low frequency HRV, while also improving their in-game behavioral performance. Critically, the breathing-based skill learning transferred to subsequent sessions in which biofeedback was not presented. Importantly, all participants remained highly engaged throughout the training. Altogether, our study showed that our VR environment can be used to train breathing regulation in an arousing and active decision-making context.

## Introduction

The work of a police officer can be seen as an evolutionary paradox: in places and situations where most people would fall prey to survival instincts of self-preservation, police officers ought to act calm, with proportionality and benevolence. As a result, working as a police officer can elicit considerable stress, with high prevalence of work-related physical injuries (West et al., 2017) and stress-related symptoms (Leppma et al., 2017) which can lead to mental disorder (Carleton et al., 2018) and suicidal ideation (Di Nota et al., 2020). Additionally, police officers are under constant scrutiny from both internal and external sources, including the public display of mistakes to ensure accountability (Skolnick and McCoy, 1984). The nature of their dangerous work as well as being under ongoing surveillance puts a great deal of pressure on police officers.

A large body of literature has shown that, besides affecting physical and mental wellbeing (Stetz et al., 2007), stress can impair decision-making aspects which are crucial to the policing job. Experimental studies have consistently shown that under high levels of arousal, decision-making becomes more impulsive and less goal-directed (Porcelli and Delgado, 2017), which is related to impaired control by prefrontal brain regions under stress (Arnsten, 2015; Maier et al., 2015). The detrimental effects of arousal have also been demonstrated in police, where high arousal has been associated with impaired shooting accuracy, as well as decreased inhibitory control (Nieuwenhuys et al., 2015; Hashemi et al., 2019). It is essential that police officers manage their stress while on the job to avoid costly mistakes such as the inappropriate use of force.

To train coping under stress in police officers, recent studies investigated the training of behavioral skills under pressure, which improved basic perceptuomotor skills such as shooting accuracy under threat (Nieuwenhuys and Oudejans, 2011) or spatial orientation (Driskell et al., 2001). However, this type of training may not always improve relevant higher-level decision-making under stress, such as when asked to make shoot/don’t shoot decisions (Nieuwenhuys et al., 2015). To counter this stress-induced performance drop and improve decision-making under threat, training efforts have moved beyond the focus on performance, by adding in police education elements to help officers directly manipulate the bodily stress response itself (Bouchard et al., 2012a; Mccraty and Atkinson, 2012; Andersen and Gustafsberg, 2016). In some cases, this opportunity for physiological modulation was even delivered intermixed with action in realistic scenario-based environments (Andersen and Gustafsberg, 2016; Andersen et al., 2018; Di Nota et al., 2021). This type of biofeedback training mainly focuses on promoting emotional regulation by helping the officer control their physiological arousal by giving feedback on their objective physical stress level (e.g., indexed by heart rate parameters, see below) to improve physiological awareness and gain more voluntary control over their physiology (Weerdmeester et al., 2020).

After studies showed the usefulness of biofeedback to enhance stress regulation (McCraty et al., 2009; Bouchard et al., 2012b) several police forces worldwide have implemented training programs in which a biofeedback training component is included. However, according to a recent survey in Netherlands, this type of training was generally negatively appraised by police officers and did not result in substantial improvements in reported stress regulation (van der Meulen et al., 2018). These disappointing results might be attributable to the fact that in current practice, biofeedback is often delivered in a passive classroom setup, which may not feel relevant to police officers and thereby affect their engagement. Yet, engagement is a key prerequisite for behavioral change (Holzinger et al., 2006). To promote both engagement and generalization to real-life stressful situations (skill-transfer), it is important to train stress regulation within a progressively more active and thereby more representative action context (Seifert et al., 2018; Staller and Körner, 2019). Indeed, just as the current biofeedback applications often lack elements to make the learning process engaging and relatable, excessive stress might also prevent learning (Di Nota and Huhta, 2019). To better reappraise stress into challenge and to improve performance under stress (Jamieson et al., 2010), it seems necessary to design a training context in which stress is kept at a moderate, optimal level that is not too low, but also not overwhelming and excessive.

One way to address previous shortcomings in classroom-based training is by creating engaging environments in Virtual-Reality (VR) where physiological control must be exerted while performing decision-making actions in an arousing context. VR is a useful tool to create controlled—yet representative—environments, allowing to elicit high levels of arousal and engagement (Parsons, 2015; Miller et al., 2019). However, re-creating a highly realistic virtual police environment in VR can also have negative consequences on subjects’ experiences due to slight mismatches with reality (Wilson and Soranzo, 2015) and the challenges in recreating genuine verbal and tactile interactions (Michela et al., 2019). To remedy this, the use of game mechanics (a set of rules and events defining the game experience) inspired by commercial videogames offers the possibility to re-create a genuine feeling of threat and immersion, moderate enough for learning to take place while at the same time boosting engagement (Allcoat et al., 2015; Cummings and Bailenson, 2016; Lin, 2017; Slater, 2018; Schoneveld et al., 2019). Game mechanics also present another advantage over realistic environments in VR, which is the ease of emotional elicitation and repeatability of the experience (Lobel et al., 2016; Michela et al., 2019; Scholten and Granic, 2019). Ultimately, gaming contexts also alleviate the emotional impact that poor performance could have on feelings of professional self-efficacy, reduces the chance of overtraining motor responses that may be less adequate in real-life and limits reactivation of potentially traumatic experiences, since the actual action is rather far removed from real policing contexts (Michela et al., 2019). Evidence-based VR trainings are, however, currently scarcely available (Di Nota and Huhta, 2019) and to the best of our knowledge, no training has been described that makes use of the aforementioned VR and gaming assets. Therefore, we developed a VR environment that offers the player the possibility to train breathing-based biofeedback skills in real-time while immersed in an engaging active decision-making game (Brammer et al., 2021).

For the physiological training component, our VR application aligns with previous training practices in Dutch Police. Specifically, biofeedback training applied to police officers has mainly focused on abdominal deep breathing (van der Meulen et al., 2018; Bennell et al., 2021), which has been shown to increase heart-rate variability (HRV) by respiratory sinus arrythmia (RSA; Hirsch and Bishop, 1981) reflecting parasympathetic nervous system dominance (Russo et al., 2017). The influence of deep breathing on the parasympathetic nervous system can be measured in both the low frequency and high frequency spectrum of HRV (Shaffer and Ginsberg, 2017; Kromenacker et al., 2018), as well as the coherence between breathing and HRV (Shaffer et al., 2014; Schwerdtfeger et al., 2020). This type of biofeedback has proven useful in a variety of training applications, from performance training for athletes (Jiménez Morgan and Molina Mora, 2017) to stress and anxiety management (Goessl et al., 2017). Importantly for police applications, an elevated HRV is also related to better performance under threat (Hansen et al., 2009), and was shown to be effectively modulated by biofeedback, hence reducing stress in contexts related to police realities (Bouchard et al., 2012a; Andersen et al., 2015, 2018; Andersen and Gustafsberg, 2016) and also improving cognitive control (Laborde et al., 2021). In our VR environment, biofeedback was provided by modulating the width of the field of view according to breathing rate and depth, thus making real-time physiological regulation key to being able to perform well in the game.

For the behavioral assessment components during the training in our VR environment, we focused on one of the key decision-making processes that is affected by stress and related to police-relevant go/nogo decisions, namely shoot/don’t shoot decisions (Nieuwenhuys et al., 2015; Gladwin et al., 2016). Impairments in response inhibition are known to be especially high when individuals are primed to believe that they will need to take action and the stakes are high (Johnson et al., 2018; Taylor, 2019). Thus, we designed our game mechanic around these processes of response inhibition under stress and the impact of priming. Having those metrics imbedded in the same environment as the physiological training allows for direct measurement of the impact that physiological training has on performance.

The first overarching goal of the current study was design validation. First, we tested if the VR environment was, as hypothesized, successful in creating a challenging environment that elicits clear increases in levels of arousal (assessed *via* heart rate) and self-reported engagement. Second, we tested if our biofeedback (a) increases slow and deep breathing and HRV, and (b) raises physiological awareness for the participants. Third we explored whether our setup allowed extraction of meaningful behavioral metrics concerning response inhibition and priming. Behavioral measurements were all extracted from the VR environment, with the auxiliary aim of documenting interactions between behavioral metrices and physiology.

The second main goal of the current study was to perform a preliminary evaluation of the game’s potential to train breathing-based biofeedback in an active decision-making context. We hypothesized that breathing biofeedback score would improve over the training, along with HRV. Moreover, biofeedback-driven physiological regulation skills should transfer to the same action context, when experienced without biofeedback. This preliminary proof-of-concept was performed by means of a withdrawal single-case experimental design (SCED), applied to a sample of nine trainers from the Dutch police, a difficult population to get access to and test due to their usual work load, yet highly valuable given that they contain all the critical insight in both the required skills for dealing with stress and the challenges surrounding teaching those skills to police recruits. These trainers took part in a ten-session training program. Withdrawal SCED has already been successfully used in investigating the potential of biofeedback (e.g., Bossenbroek et al., 2020). This design has the advantage of providing rich datasets to investigate the dynamical evolution of skill acquisition within and across sessions (Smith and Little, 2018), and inform future research about the often overlooked aspect of minimal training length required for efficient training (Di Nota et al., 2021).

## Materials and Methods

### Participants

Participants were nine male police trainers with an average age of 43.2 years (SD = 6.45) and with an average of 18.4 years (SD = 8.6) of operational background as a police officer. Their average trait anxiety was 27 (range 23–34 on a scale of 20–80), which indicates participants in our sample to be non-anxious. Only three participants reported playing video games in their free time, to a maximum of 4 h a day during weekends. Participants were recruited from a Police skill training center, in Netherlands, hereafter referred to as IBT center (“Integraal Beroepsvaardigheids Training centrum”). Given that this was a proof-of-concept study, the number of participants was based on earlier studies using similar SCED methodologies (Ebbinghaus, 2005; Smith, 2012; Smith and Little, 2018; Bossenbroek et al., 2020). Participation was voluntary and handled by the coordinator at the IBT center. According to the rules of the Dutch Police, financial compensation of the police officers functioning as participants was not allowed. Therefore, for each participant a donation of 50 euros was allocated to a fund for the training of “PTSD dogs” (Actie ZeeHond, n.d.). The research procedures were approved by the ethical committee of the Faculty of Social Sciences of Radboud University Nijmegen (ECSW-2020-112). All participants provided informed consent in writing prior to participating in the study, in line with the guidelines of the Declaration of Helsinki (WMA, 2018).

### Materials

#### Physiological Recordings

Participants’ breathing rate was measured using a respiratory inductance plethysmography (RIP) belt from Plux S.A. and a BITalino (r)evolution board (Batista et al., 2017). The heart rate of the participant was recorded by a Polar H10 chest strap, which extracts R–R intervals (i.e., the time between consecutive R-waves of the QRS electro cardiac signal). Both physiological recording units broadcasted their data to a Raspberry Pi 4 Model B, which was also used to calculate the breathing biofeedback scores with custom-made python software, based on the open source EEGsynth library (Brammer et al., 2021; Oostenveld, n.d.).

#### Virtual-Reality Material, Model, and Task

An HTC Vive setup was used to immerse the participants in a virtual environment that consisted of a poorly lit underground parking garage. The VR trackers were set 3–4 m apart, giving the player a minimal play area of 4 square meters. One of the two VR controllers was wrapped by a 3D printed case, giving the controller the weight and shape of a gun. The second VR controller was attached to the vest of the participant and used as a dispatch-radio in the game.

##### Operationalization

The VR environment was designed to incorporate game mechanics based on existing experimental laboratory tasks, as shown in Table 1. The paradigms listed were incorporated as they represent specific behavioral aspects known to be affected by stress and relevant in the decision-making processes. The first paradigm is emotional regulation, in our case physiologically influenced through breathing-based biofeedback presentation. Its implementation as a modulation of the width of the field of vision makes the biofeedback relevant for the player, since in active contexts it is easy to ignore the biofeedback if it does not interfere with action. Modulating the peripheral view directly impacts the difficulty of detecting approaching enemies in the VR environment.

**TABLE 1:** Operationalization of in-game tasks.

Experimental paradigm	In-game operationalization	In-game mechanic	Game output / proximal measure	Training outcome
Emotion regulation	Biofeedback	Width of the field of view linked to breathing pace	Breathing control and HRV scores	Physiological control
Response inhibition (Go/NoGo)	Shoot/don’t shoot	Shooting at targets matching dispatch information	Accuracy	Response control
Priming	Dispatch bias task	Targets’ (mis)match with dispatch information (body shape; eye color)	Accuracy based on body type of the target	Bias resistance

*Model for the incorporation of experimental paradigms in the game environment; HRV, heart-rate variability.*

The second and third paradigms were selected based on existing literature investigating police performance in decision making contexts. Specifically, response inhibition under stress, operationalized as go/nogo (shoot/don’t shoot) decision-making, has been linked to increased error-rates with increased threat level (Nieuwenhuys et al., 2015; Hashemi et al., 2019, 2021). Similarly, priming has been shown to increase wrong shooting decisions in police officers primed with a radio dispatch indicating that an upcoming opponent was armed (Taylor, 2019). These two paradigms were implemented in the game in the form of a zombie shooting task (see below).

#### The Virtual-Reality Game

The game scenario unfolded as follows: Participants found themselves in a dimly lit parking garage, received dispatch information about hostile targets with a description of their features, were approached by (friendly and hostile) targets, and decided whether to shoot or not. Each game session lasted approximately 15 min. Targets were coming toward the participants from all directions, in fourteen waves. During each wave, the participant would receive dispatch information over the walkie-talkie to shoot the hostile but not the friendly targets, including a description of the hostile targets. All targets had two identifying features that made them recognizable as friendly or hostile. The *large identifier* was their body type (tall male, small male, tall female, or small female). During the game this identifier became less reliable to identify a target, as hostile targets had increasingly more varying body types (starting with 100% targets matching the description and decreasing to 50% in the last waves). The *small identifier* was their eye color (red, blue, or yellow); this was always 100% reliable. To summarize: The dispatch information for the eye color of hostile targets would always be correct, but the dispatch information for their body types was not completely accurate. The body type was visible from a far distance, while eye color was only visible when the target had approached to a close distance. Hence, identifying targets with the correct eye color and shooting at them on time was our implementation of the go/nogo task component, while the radio dispatch announcing suspected body types associated with the targeted eye-color was our implementation of the priming component.

Each time the player shot, the game recorded if the shot was a hit or a miss. In case of a hit (i.e., hostile target with correct eye color), the game logged the body type of the target and the distance between the target and the player. Shooting a friendly target (i.e., wrong eye color) was punished by a loud burst of noise. Each time a target reached the player without being shot, the game logged the body type of the target, whether it was hostile and the time that it had spent in the line of sight of the player. Hostile targets would then stay next to the player and attack them until shot. The player was notified of the attack by sound, and a red halo appeared framing their field of view. Players could not “lose” the game in the sense that they reached game-over, the hostile targets had to be all shot before the next wave would start. Please note, in contrast to more traditional laboratory assessments of go/nogo performance, different trial types were here presented at the same time (i.e., multiple targets approached the player at the same time). Hence, time pressure was created by the fact that multiple choices had to be made concomitantly, to simulate a short response window, required to maximize false alarm rates (Young et al., 2017). The maximization of false alarms was needed to provide enough improvement margin to the player and benefited from the non-realistic environment to mitigate risks in terms of professional self-efficacy in players.

At the end of each session, the participant was presented with scores ranging from 0 to 100%, summarizing their performance on three metrics commonly used to describe police performance in the field: Control over the situation, control over the suspect and control over the self (Binder and Scharf, 1980; Huhta et al., 2021; see Supplementary Material 1 for the formulas used to calculate these scores). While the first two scores represented behavioral elements, the last one was a summary of the breathing pace performance rewarded by the biofeedback. The scores were calculated to make the scores of “control of the situation” and “control of the suspect” relatively easy: Participants could achieve high scores without excessive effort. On the other hand, the “control of the self” score directly represented the physiological control score, thus helping to nudge the players toward focusing on physiological control more in the following sessions.

#### Biofeedback Parameter and Implementation

**FIGURE 1:**
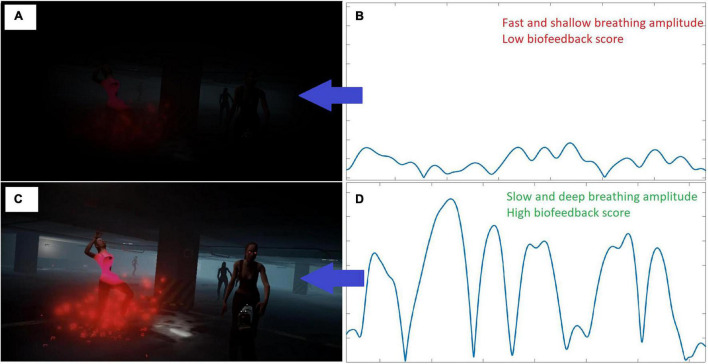
Implementation of the biofeedback as peripheral vision modulation. The quality of the vision (on the left) was linked to the biofeedback score, calculated from the breathing speed and amplitude (on the right); **(A)** Vision with a biofeedback score equal to 0 (the player is not breathing at the rewarded pace); **(B)** example of fast and shallow breathing amplitude, corresponding to a breathing biofeedback score of 0; **(C)** Vision with a biofeedback score equal to 1 (maximum adherence to rewarded breathing pace) or in A-phase sessions in which online biofeedback was not presented to the player; **(D)** example of slow and deep breathing amplitude, corresponding to a breathing biofeedback score of 1.

In sessions in which the biofeedback was displayed to the player (ABBABABABA withdrawal design; A = without biofeedback: session 1, 4, 6, 8, and 10; B = with biofeedback: session 2, 3, 5, 7, and 9), a breathing pace of eight breaths per minute was rewarded by having optimal vision, with faster or slower breathing paces being progressively punished by reduced (tunneled) vision (see Figure 1).

The visual feedback on participants’ breathing pace was implemented by reducing the vision of the player in the VR task proportionally to their non-adherence to the rewarded breathing pace. This “tunneled” vision served to help the player control their breathing pace in the heat of the moment, when task demand reduced awareness of their own physiological processes. Specifically, the biofeedback value rewarded slow and deep breathing by promoting high amplitudes of breathing paces in the 6–10 breath per minute range. It was calculated by performing spectral density estimation on the incoming breathing signal and then calculating the power of the signal within the target frequency range divided by the power outside the target frequency range (c.f., Brammer et al., 2021; Supplementary Figure 2). Please note that the power was calculated as area under the curve, thus reflecting the amplitude (depth of breathing) within the target range. This value was updated at a rate of 0.5 Hz, and ranges between 0 and 1. Hence, every 2 s, a breathing segment of the last 30 s of breathing data was analyzed, resulting in an overlap of 28 s between consecutive segments. It rewarded diaphragmatic breathing at a pace of eight breaths per minute. This target is faster than the six breaths per minute pace recommended by the literature to increase HRV (Hirsch and Bishop, 1981; Brown et al., 1993; Ben-Tal et al., 2014), as piloting revealed that such a low pace was too hard to achieve in action. The exact signal processing pipeline and data extraction can be found in the Supplementary Material of our previous article, detailing the development of the biofeedback parameter (Brammer et al., 2021). Importantly, the visual presentation of the biofeedback could be switched off, in which case the player would always have a full vision, independently from their breathing biofeedback score. This parameter was experimentally manipulated between training sessions to test the impact of biofeedback presentation (see section “Procedure”).

#### Questionnaires

All questionnaires were administered in a paper and pencil form. The following questionnaire data were collected.

##### Trait-Assessments of Anxiety

Participants’ trait anxiety was assessed in the first session using the Dutch State-Trait Anxiety Inventory with twenty items and four response options (1 = “almost never” to 4 = “almost always”; van der Ploeg, 1984). An example of a statement is “I feel satisfied with myself.” A total score was calculated by calculating the sum of all item scores (range 20–80), with higher scores corresponding to less anxiety.

##### Prior Gaming Experience

Participants’ prior gaming experience was assessed in the first session using two self-constructed questions. Participants were asked how many hours a day they played video games on a weekday and how many hours during a day on the weekend. Response options were: (1) I do not play video games; (2) less than 1 h per day; (3) one to 2 h a day; (4) two to 3 h a day; (5) three to 4 h a day; (6) more than 4 h a day.

##### Pre-test Questionnaire

The pre-test questionnaire (target approach analysis, see Supplementary Material 2) is a short self-constructed questionnaire inspired by questionnaires used to investigate plan-making in real-life policing situations (Adang and Timmer, 2005), administered before each VR session to the players. They were asked to indicate which one of the three control scores mentioned above they would focus on, what scores they expected to achieve as well as the scores they expected their colleagues to achieve. An open question concluded the questionnaire, to ask the player if they had a specific strategy in mind for the upcoming session. The aim of this questionnaire was twofold: Measuring the participants’ training intentions and prime them to keep in mind the policing goals of the training endeavor.

##### Post-test Questionnaire

After each session, the post-test questionnaire (after action report, see Supplementary Material 2), a short self-constructed questionnaire, was administered to the participants. The players were asked to indicate which score they thought they had achieved, on a scale from 0 to 100%, for each one of the 3 control scores (control of the situation, of the suspect and of self) and several open-ended questions to make them think about their performance. The aim of this questionnaire was twofold: Measuring the participants’ performance estimates, and maintain awareness of the policing goals of the training endeavor. Of the three self-rated control scores, only the “control of the self” score was used for further analyses, where it was contrasted to the actual “control of the self” score achieved by the participant.

##### Threat and Challenge Appraisal

Participants’ appraisal of threat and challenge during the game was assessed after each session on an eleven-item scale developed by Mendes et al. (2007), with seven response options per item (1 = “totally disagree” to 7 = “totally agree”). Six items were indicative of the threat aspect (i.e., “this task is demanding,” “… is stressful,” “… is distressing,” “… is threatening”; “I am uncertain how I will perform”; “this task requires a lot of effort”). In addition, five items were indicative of perceived positive challenge (i.e., “I have the abilities to perform well,” “I have the expectations to perform well,” “performing well is important to me,” “this task is a positive challenge,” and “I am the type of person who does well on these tasks”). Two distinct scores (ranging from 1 to 7) were calculated for the threat and the challenge aspect, by averaging the related item scores.

##### Engagement

Participants’ engagement was assessed after each session using seven items from the Intrinsic Motivation Inventory with seven response options (1 = “totally disagree” to 7 = “totally agree”) (McAuley et al., 1989). An example statement is: “I would describe this activity as very interesting.” The negatively formulated statements (“I thought this was a boring activity” and “This activity did not hold my attention at all”) were recoded. A total score was calculated by averaging the scores on all items (range 1–7). The higher the score, the higher participants’ engagement during the training.

#### Procedure

After giving written informed consent and filling in the questionnaire about gaming experience as well as the trait anxiety questionnaire on the first session, each session consisted of participants putting on the respiration belt and the heart-rate belt, receiving a police vest, the controllers (radio and gun in the VR environment), headphone, and VR headset. Next, participants filled in the pre-test questionnaire, standing, while their physiological baseline was measured. The participants then performed the VR task for around 15 min until they completed all the waves of the game. In the first session, participants first played a tutorial in which they were instructed about processes such as confirming a radio message, followed by the actual VR task. The tutorial was also present in the second session, with additional information about biofeedback control and a breathing pace training. From session two onward, participants were instructed to breathe 5 s in and 5 s out while playing. After playing each session, participants filled in the rest of the questionnaires regarding their appraisals of the game. When the participant finished all questionnaires, they were presented with their average session scores of control over the situation, suspect and self.

To allow statistical inferences in small sample sizes, in this single case experimental design (SCED) study participants were invited ten times to the training sessions. A withdrawal ABAB design was used in which the experimentally withdrawn variable was the presence of online biofeedback on the participant’s breathing pace, where slow and deep breathing was rewarded. The online biofeedback was presented to the participants by means of vision impairment while performing the VR task. Over the course of 1 month (September 2020), all participants completed the ten sessions (phases) following a withdrawal design (ABBABABABA). The majority of the sessions were separated from each other by one to 5 days. Due to participant scheduling limitations, some sessions had, however, to be done in the same day. In such a case, the first session always was an A phase, to prevent short term carry-over from the biofeedback presentation, displayed in B phases. In other words, we wanted to prevent the risk that players would apply the breathing technique in an A phase just because they had to apply it in the B phase that happened only minutes before. This security measure was implemented to ensure that looking at contrasts between a biofeedback B phase and a subsequent A phase would reflect as much as possible learning transfer and not mere short-term habituation. Continuous breathing-based biofeedback was added in the B-phases and withdrawn in the A-phases. The design is in line with the guidelines for small-N designs, by allowing a minimum of four repetitions of the addition-withdrawal procedure (Kratochwill et al., 2013).

#### Data Preparation

Any identification information was removed from participant data and the data were securely stored on password-protected servers hosted by the Radboud University. The data was only accessible for approved members of the research team. The research data was not shared with the police organization, nor with the IBT center from which participants were recruited.

##### Physiological Recordings

The physiological data (breathing biofeedback score and inter-beat “R-R” intervals) were automatically saved at a rate of 0.5 Hz. The data points were then synchronized with the game events and averaged before the start of the game to constitute the baseline. Since the baselines were inconsistent in length, the shortest recording (29 samples = 56 s) was used as a length reference, hence we only considered the last 29 samples portion for longer baselines. Per session, the inter-beat (R–R) intervals were interpolated to extract low (0.04–0.15 Hz) and high (0.15–0.4 Hz) frequency HRV, the low/high frequency ratio of HRV, and the coherence between the low frequency HRV and breathing. The coherence score was calculated by quantifying the similarity of the breathing and inter-beat interval time-series (Shaffer and Ginsberg, 2017). This quantification, bound between 0 and 1, is frequency specific. We therefore only considered the low-frequency HRV range as it is the range at which paced breathing would happen. The low frequency coherence metric is extracted as an index of relaxation, as Hayano and Yuda (2019) suggested that breathing induced fluctuations in the low frequency spectra of HRV reflects the presence of resting function. The scripts used to extract those HRV metrics can be found on GitHub (Brammer, 2020) and were taken from the guidelines proposed by Shaffer and Ginsberg (2017).

##### Decision-Making and Response Inhibition Behavior

In-game events and actions were summed within a session to compute accuracy and signal detection measures. Hit scores were calculated by adding events where the participant shoots at an incoming hostile target before it reaches the player. Similarly, miss scores were the sums of hostile targets reaching the player before being shot, correct rejection scores were the sums of friendly targets reaching the player unharmed and finally false alarm scores were the sums of friendly targets being shot by the player. For each of those scores, we also recorded if the target involved in the event had the primed large identifier (body type announced by the radio dispatch as potentially hostile).

#### Data Analysis

##### Missing and Excluded Data

Due to a technical issue with the heart rate belt recordings, the HR data of session 1 was missing for participants 3 and 5, although the participants did complete the training session. Moreover, due to material failure with the breathing belt used for biofeedback, the breathing data of session 8 was missing for participant 4, although the participant completed the full training session. Subject 9 was excluded from HRV-related analyses, since his high frequency component of HRV was more than 3 standard deviations higher than the rest of the group, for several sessions; this was due to a lack of accuracy in the R peak detection from the heart rate belt. This problem did not affect biofeedback measurements.

##### Environment Design Validation

Due to the small sample size, this section of the results is purely descriptive, as only the biofeedback data allowed to make meaningful statistical inferences thanks to the withdrawal (ABBABABABA) design.

##### Game Arousal, Challenge, and Engagement

To descriptively assess, for each subject, the evolution of the level of arousal, challenge and engagement, individual trajectories were plotted alongside group average. For the HR, average baseline and in-game session scores were obtained. The in-game scores were obtained by subtracting the average baseline score from the in-game scores.

##### Biofeedback Relevance

To assess whether our biofeedback manipulation was successful in influencing HRV, the average breathing biofeedback score within a session was correlated with the low and high frequency components of HRV and with the coherence between the breathing and low frequency HRV. The latter correlation is used to measure the extent to which low frequency HRV is influenced by slow and deep breathing, an indicator of resting function rather than sympathetic dominance (Hayano and Yuda, 2019). Since correlations were based on all 10 sessions of eight participants in total, where each session is a data point, we used a repeated measure correlation approach using the R package “rmcorr” (Bakdash and Marusich, 2017, 2021). Subject 9 had to be excluded from those analyses due to a measurement error.

To measure how awareness of the breathing control performance evolved over the course of the 10 sessions, we computed for each session the difference between participants’ self-rating in breathing performance (an auto evaluation ranging from 0 to 100%) and the actual session score of “control of the self” (the percentage of the session that the player spent with a breathing biofeedback score of at least 0.8 over 1). Thus, the awareness score was calculated as: real “self-control” score—self rating “of self-control” score.

To further investigate the relevance of biofeedback, we looked at how often players mentioned breathing (e.g., “I need to focus more on the breathing”), biofeedback visual impairment (e.g., “Too much tunnel vision”) and self-control score (e.g., “Focus on self-control”) in the open-ended questions asked to the player before and after the VR task. Mentions of action-related elements (e.g., “Reload more often” or “Monitor 360”) were scored as well.

##### Decision-Making Behavior

To measure decision-making (shoot/don’t shoot) performance, we calculated the amount of hits, misses, correct rejection and false alarms per subject and session. Additionally, sensitivity {*d*′ = [z(*Hitrate*)−z(*Falsealarmrate*)]} and response bias {criterion = -[z(*Hitrate*)−z(*Falsealarmrate*)]/2} were computed according to signal detection theory (McFall and Treat, 1999). Since some participants managed to avoid false alarms for an entire session, a loglinear correction was applied to the data to avoid infinite values (Hautus, 1995; Stanislaw and Todorov, 1999). To describe the effect of the priming, we calculated per session and subject the difference in false alarm rate between targets that had the body type primed by radio dispatch and those who did not^1^.

##### Training Efficiency: Physiological Control

Next, we tested the link between the presentation of online biofeedback in B-phases and the improvement in breathing control in action witnessed in all subjects. In this methodology, the goal is to search for the effect of repeatedly adding and removing a variable. Evidence for a causal effect is then gathered through multiple analyses to ensure that the found effect is genuine. In our case, the experimentally added and removed variable is the presence of biofeedback, implemented as the tunneled vision described above. According to the requirements, of the SCED methodology (Kratochwill et al., 2013), multiple datapoints have to be extracted per session, which allows this section of the study to perform inferential statistics. The biofeedback values of a session were separated in 15 s bins and averaged for each bin. The biofeedback scores of individual participants were analyzed within and between sessions (A-sessions, B-sessions, B-A-sessions, and A-B sessions). The six features of the SCED visual analysis were level, trend, variability, immediacy of effect, consistency of data patterns across similar phases and degree of overlap of data (Kratochwill et al., 2013). A change of the data-patterns when there was a change in condition (addition or removal of biofeedback) indicates the biofeedback had an effect. To determine whether there was a significant intervention effect for a participant, a minimum of three such changes was needed for at least three of the six features (Horner et al., 2005; Kratochwill et al., 2013; Lane and Gast, 2014). Additional descriptive representations of HRV parameters were included in this section, since the main goal of breathing-based biofeedback was to calm participants by regaining parasympathetic dominance through HRV increase.

##### Single-Case Experimental Design Analysis

To illustrate the within and between session dynamics, the evolution of the biofeedback scores, evaluated at the 15 s bin level throughout the entire training, were plotted for each subject along with separated fitted trend lines for the A and the B phases. The trend lines were obtained with the MATLAB function “polfit.m.” To increase visibility of the data, the lines were smoothed with the moving average option of the MATLAB function “smooth.m.” Since the biofeedback score was measured on a time window of 30 s, the first two data bins of each session were discarded to avoid analyzing data from the baseline period.

Of the six SCED features used to test the role of biofeedback, data overlap is the one presented in the result section as it is most relevant to our design. For completeness, we report other aspects in the Supplementary Material 3. To test whether vision impairment was a salient enough way of providing biofeedback in B-sessions, and causally influenced the player’s behavior, data overlap of the biofeedback values of consecutive sessions was calculated by using the Kendall’s tau ranked correlation coefficient (Kendall, 1938). For each subject, the tau scores were then aggregated into two distinct scores (addition and removal) by combining effects of single contrasts (Manolov et al., 2015; Tarlow, 2017a,^2018^). The addition score encompasses the tau scores for A-to-B phases contrasts, hence when comparing consecutive sessions where the first is played without online biofeedback and the second with biofeedback. Conversely, the removal score encompasses the tau scores for B-to-A phases (transition from with to without biofeedback). Kendall’s tau was extracted by using an edited version of the R code by Tarlow (2017b). The editing consisted in removing Theil–Sen estimator used for baseline trend correction (Tarlow, 2017b). Baseline trend correction was not used for this dataset, as trend analysis revealed that a within session trend was often present but negative, hence correcting for it would lead to false positive results.

## Results

### Goal 1: Environment Design Validation

#### Does the Virtual-Reality-Game Environment Evoke Arousal and Engagement?

**FIGURE 2:**
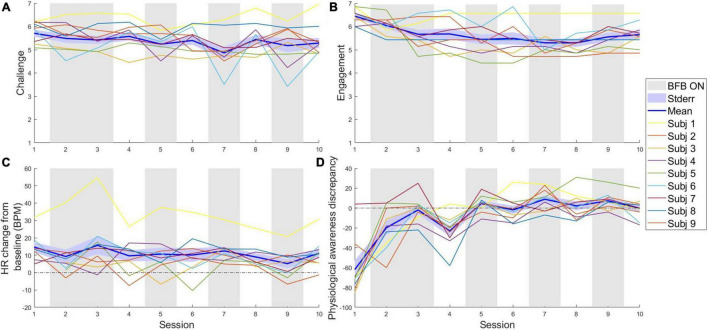
Stability of arousal and engagement during the course of the training. Changes across the whole training in: **(A)** self-reported challenge (1 = very low to 7 = very high); **(B)** mean engagement (1 = very low to 7 = very high); **(C)** HR change, in BMP, from the 30 s pre-test baseline (the dashed line represents a null change); **(D)** Difference between the self-reported estimation of biofeedback control score after the VR task and the real score (range 0–100%; plotted value = real score—reported score). Stderr, standard error of the mean; BFB ON, Sessions in which online biofeedback was presented to the participants.

The VR-game elicited psychophysiological arousal and was experienced as challenging and engaging in the small sample at our disposition. This is indicated by the self-reported threat and challenge appraisals, the increase in HR and the self-reported engagement score (see Figures 2A–C). As expected, police officers reported consistently low levels of experienced threat, with an average of 2.39 on a 7 point scale (SD = 0.86; see Supplementary Material 4) over all sessions and participants. However, challenge scores were consistently high (Figure 2A) indicating that the participants experienced the game as positively stimulating (*M* = 5.36, SD = 0.65). In terms of absolute HR during the sessions, the average was 79 BPM. There was a marked peak in HR during the first session (*M* = 99 BPM) with substantial variation between subjects from *M* = 78 BPM (for subject 4) to *M* = 176 BPM (for subject 1). This strong variability was only witnessed for the first session. Relative to the baseline period immediately preceding the game start, an average increase of HR from baseline of approximately 10 BPM was observed (Figure 2C), similar to increases witnessed in established stress induction protocols (e.g., see Vogel et al., 2015). As shown in Figure 2B also participants’ reported engagement (scale 1–7) was generally high (M = 5.65, *SD* = 0.66), steadily decreasing until session seven, where the average engagement was still 5.3, corresponding to a moderately high level of engagement.

#### Does the Biofeedback Implementation Influence Heart-Rate Variability and Facilitate Physiological Awareness?

In our limited sample, a strong and significant positive relation was found between the average biofeedback score and the coherence between low-frequency HRV and respiration in a session [r(68) = 0.72, *p* < 0.001]. A smaller positive correlation was also found for low frequency HRV [r_*s*_(68) = 0.47, *p* > 0.001] but not for high frequency HRV [r_*s*_(68) = 0.15, *p* = 0.212]. Hence, biofeedback scores correlated strongly with breathing induced fluctuations in HRV, a resting function index (Shaffer et al., 2014; Hayano and Yuda, 2019).

To evaluate the participants’ self-awareness of physiological control during the training process, the differences between the self-rating of physiological control and the objective biofeedback score are plotted in Figure 2D. A fast reduction in differences (overestimation) can be seen in the first half of the training process, with a notable yet short-lived increase in difference once online biofeedback was removed for the first time in session 4. Thus, subjects became considerably more accurate in their assessment of their physiological control over the course of the training.

Additionally, the answers given to the pre- and post- questionnaires (see Supplementary Material 5), indicated that the participants mentioned breathing more after biofeedback sessions, both in the pre-test phase before the session (from five subjects in session 1 to all nine subjects in session 3) and in the post-test phase when debriefing (from four subjects in session 1 to eight subjects after sessions 2 and 3). Thus, biofeedback sessions successfully increased attention to breathing control.

#### Does the Behavior in Our Application Follow Expected Patterns Relating to Response Inhibition Under Stress?

**FIGURE 3:**
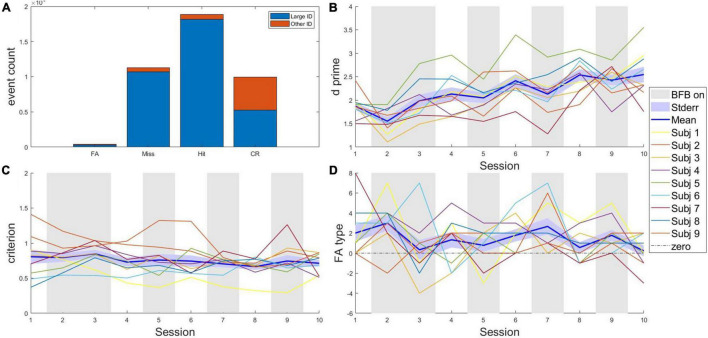
Behavioral metrics (in-game). **(A)** Go/nogo action distributions across sessions and subjects. Columns are sub-divided according to the large identifier of the target related to the trial. **(B)** *d’* from signal detection theory indicating sensitivity to target type (hostile/friendly); **(C)** criterion from signal detection theory indicating potential changes in strategy **(D)** Difference score of false alarm depending on the body type (primed body type—unprimed body type); Hit, hit; CR, correct rejection; FA, false alarm; Miss, misses; large ID, has the primed body type, announced as presumably hostile in radio dispatch; other ID, does not have a body type announced as presumably hostile in radio dispatch; BFB on, Sessions in which online biofeedback was presented to the participants; Stderr, standard error.

The distribution of correct and incorrect decisions in the game is presented in Figure 3 (see Supplementary Table 6.1 in Supplementary Material 6 for details). Results showed that misses were by far the most likely type of mistake and police trainers avoided to make false alarm responses (Figure 3A). Over time, participants steadily increased in accuracy (sensitivity) as assessed by d prime (*d*′; Figure 3B), while their response bias (criterion) remained stable and conservative (Figure 3C). Interestingly, *d*′ tended to be lower in sessions with biofeedback. Lastly, we investigated dispatch priming. As shown in Figure 3D, a higher proportion of friendly targets being shot (FA) was composed of targets whose large identifier (body type) was announced as presumably hostile in radio dispatch.

### Goal 2: Training Validation

#### Biofeedback Score

**FIGURE 4:**
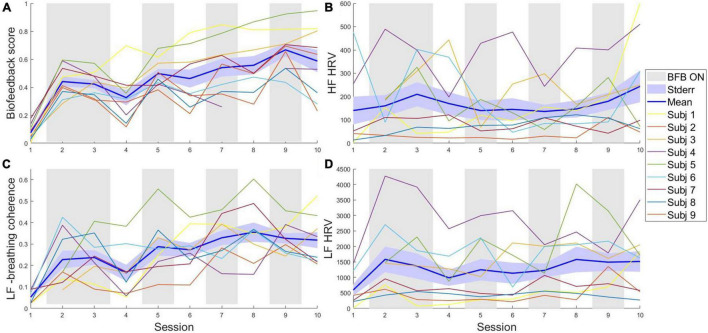
Evolution of the breathing biofeedback score and the HRV indexes. Changes across the whole training in panel **(A)** the average biofeedback score; **(B)** the high frequency component of HRV (0.15–0.4 Hz); **(C)** the coherence between the low frequency HRV and breathing; **(D)** the low frequency component of HRV (0.04–0.15 Hz). BFB ON, B phases, where online biofeedback was presented; Stderr, Standard Error; LF-breathing coherence, coherence between the low frequency component of HRV and breathing; LF HRF, low frequency HRV.

The average biofeedback scores per participant are depicted in Figure 4A. The overall biofeedback score was *M* = 0.077 (SD = 0.16) in the first session (A_1_), increasing to *M* = 0.497 (SD = 0.279) across all sessions with online biofeedback (B phases) and to *M* = 0.460 (SD = 0.266) across the subsequent sessions without online biofeedback (A_2–5_ phases). Six participants (2,4,6,7,8 and 9) showed higher biofeedback scores in B-phases (sessions with online-biofeedback), whereas three participants (1,3 and 5) had a higher average biofeedback score in A-phases (see Supplementary Material 3 for details).

#### Evolution of Heart-Rate Variability

We here illustrate how beneficial the breathing-based biofeedback training is to influence HRV through RSA modulations. In Figures 4B–D the evolution of the low and high frequency components of HRV, and the coherence between low frequency HRV and the breathing pace are presented. While the low frequency HRV-breathing coherence (Figure 4C) parallels the increase over time shown by the breathing biofeedback score, displayed in Figure 4A, quite accurately, this pattern is less apparent for low (Figure 4D) or high (Figure 4B) frequency HRV. This indicates that our operationalization of the biofeedback parameter as a paced breathing reward successfully promoted higher coherence between the breathing and the HR (Shaffer et al., 2014; Schwerdtfeger et al., 2020), an index of resting function (Hayano and Yuda, 2019).

#### Single-Case Experimental Design Analysis

**FIGURE 5:**
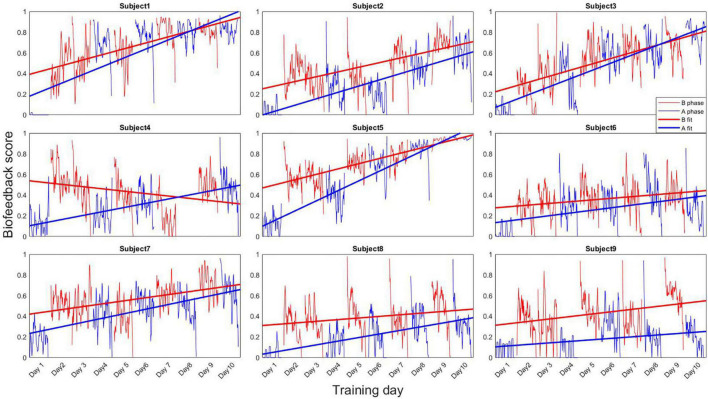
Evolution of the breathing biofeedback score across the entire training period. Each datapoint represents the average biofeedback value for a 15 s epoch. Scores range from 0 (no adherence to the rewarded breathing pace) to 1 (perfect adherence to the rewarded breathing pace). B phase, breathing score for B phase sessions (Session 2, 3, 5, 7 and 9); A phase, breathing score for A phase sessions (Session 1, 4, 6, 8 and 10); B fit, fitted line for the B phase sessions; A fit, fitted line for the A phase sessions.

The within and between session evolution of each participant’s breathing biofeedback score (indicative of breathing control) is presented in Figure 5. Trend lines revealed that every subject showed a positive trend with increasing breathing biofeedback scores over time for both A and B phases, except subject 4 who showed no improvement in the B phases (red lines). Subjects 1, 3 and 5 displayed a particularly steep learning pace. Interestingly, those are also the subjects with a higher biofeedback average in A-phase sessions than in the B-phase ones.

##### Data Overlap

To assess the influence of the breathing biofeedback presence (on or off), changes in breathing biofeedback score were related to the addition or removal of biofeedback by means of Kendall’s tau (Table 2). Results indicated a consistent positive effect of the addition of online biofeedback across subjects (A to B phase). Out of 36 individual transitions where biofeedback was added, 25 transitions were found to be positive and significant, with four subjects (2, 5, 8, and 9) each having three significant effects of non-overlap and a significant meta-effect when all A-to-B contrasts were merged, thus reaching formal criteria for a significant intervention effect. Importantly however, all nine subjects showed an effect in the same positive direction (Table 2). This suggests that, while there was a difference in the robustness of learning, participants generally improved in physiological control when online biofeedback was presented. Upon removal of biofeedback (B to A-phase), the pattern was more mixed. Out of 36 removal transitions, 14 transitions were found to be significantly negative. Only subjects 8 and 9 showed three significant repetitions of non-overlap, and only subject 9 had a significant meta-effect of withdrawal with in total six out of nine subjects showing a negative effect. Overall, the results support a causal effect of the biofeedback experimental manipulation, with seven participants (1, 2, 5, 6, 7, 8, and 9) showing a minimum of three significant non-overlap effects in the expected direction (Kratochwill et al., 2013). More elaborate SCED analyses can be found in the Supplementary Material 3, which according to formal SCED guidelines together indicated a moderate positive effect of our training procedure (Kratochwill et al., 2013).

**TABLE 2:** Kendall’s tau non-overlap indices for consecutive sessions.

Subject	Kendall’s tau									
	Addition (A-to-B phase contrast)					Removal (B-to-A phase contrast)				
	S1-S2	S4-S5	S6-S7	S8-S9	Meta	S3-S4	S5-S6	S7-S8	S9-S10	Meta
1	0.8***	−0.13	0.21**	−0.01	0.26	0.21**	0.27***	−0.16*	0.03	0.09
2	0.67***	0.16*	0.47***	0.29***	0.42*	−0.09	−0.3***	−0.12	−0.1	−0.15
3	0.53***	0.25***	0.08	0.06	0.25	−0.04	0.02	0	0.17*	0.04
4	0.6***	0.35***	−0.23**	*NaN*	0.26	−0.4***	−0.12	*NaN*	−0.05	−0.19
5	0.62***	0.37***	0.17*	0.35***	0.40*	−0.26***	−0.01	0.1	0.17*	0.004
6	0.52***	0.14*	0.11	0	0.21	−0.04	−0.17*	0.06	−0.24**	−0.09
7	0.47***	−0.03	0.12	0.29***	0.22	−0.05	0.13	−0.17*	−0.09	−0.04
8	0.62***	0.39***	0.25***	0.22**	0.39*	−0.34***	−0.3***	−0.02	−0.24**	−0.22
9	0.56***	0.61***	−0.02	0.49***	0.43**	−0.43***	−0.19*	−0.23**	−0.55***	−0.35*

*p < 0.05; **p < 0.01; and ***p < 0.001.

*Gray cells indicate significant non-overlap between two consecutive sessions; i.e., S1-S2 = contrast between the first and the second session (the same applies for the other headings).*

*Meta, Overall effect, obtained by merging the effect-sizes of the single contrasts.*

## Discussion

We aimed to validate the design of a novel biofeedback training tool for in-action physiological regulation in police. Our first goal was design validation. We found that the VR game was successful in creating an engaging, challenging, and arousing environment. Our in-game biofeedback implementation succeeded in improving physiological awareness. Moreover, behavioral metrics of performance indicated suitability for probing response inhibition and priming effects under stress. For our second goal, investigating the training effectiveness, we additionally found support for the trainability of deep-breathing by presenting biofeedback in-action. These results, while preliminary given the small sample, suggest the feasibility and promise of influencing physiological control in an active decision-making context with tools like our new VR biofeedback application.

One of the rationales for using a VR context to train physiological control was the possibility to evoke strong emotions (Parsons, 2015). As expected, self-reports indicated a high sense of positive challenge (see Figure 2A and Supplementary Material 4), but a low sense of threat. This result indicates that the game is a good learning environment as it is not perceived as too threatening (Jamieson et al., 2013), although this interpretation should be cautious given the tendency of police officers to underreport socially undesirable emotions (McCanlies et al., 2014; Habersaat et al., 2021). One additional possible explanation for the low threat score, as pointed out by Weerdmeester et al. (2020), is that the high level of challenge experienced by the participants throughout the training could partially be explained by the effect of biofeedback, which has been theorized to help participants better appraise threat into challenge since interoceptive signals of stress are dampened.

In terms of arousal, elicitation was successful in our VR environment as in-game HR increases from baseline were comparable to established stress induction protocols (see Figure 2C; Boesch et al., 2014; Vogel et al., 2015). However, we cannot establish the extent to which this arousal is of emotional nature or due to movement, as players were moving more during the game than at baseline. Yet, research by Gorini et al. (2011) showed effects of similar magnitude from exposure to a VR environment without participant movement.

We used VR to maximize the sense of engagement experienced by the participants, as it has been highlighted as an important non-specific factor fostering behavioral change (Holzinger et al., 2006). Engagement also helps with accurate recollection of the participant’s physiological arousal (McCall et al., 2015), thus improving biofeedback learning. The high and lasting engagement in our sample (see Figure 2B) may be ascribed to the advantages of VR (Riva et al., 2007), but could also be partially explained by peer pressure to perform, as our participants were, on their own initiative, actively comparing their performance scores with each other, a common practice in the police forces (Chen et al., 2013). This behavior provides support to the idea that reporting police-relevant scores at the end of each session helps strengthen engagement.

Just as the scores helped the participants to engage in the training, it also helped them to better estimate their performance and enhance physiological awareness, as illustrated by the improvements in physiological scores estimation (see Figure 2D). Similarly, the integration of biofeedback as peripheral view modulation was successful in eliciting awareness of breathing, when evaluated by self-report in open-ended questions (see Supplementary Material 5). This “tunneled vision” is known to be a relatable phenomenon for police officers (Dirkin, 1983; Klinger and Brunson, 2009). Indeed, as shown by the participant’s written reports before and after action, mentions of breathing-related elements increased throughout the training.

Regarding the behavioral metrics, despite the small sample, we could show preliminary evidence that behavioral measurements were suitable for individual performance indexes to be extracted, per session (see Figure 3). It is worth noticing that decision-making stably improved throughout the training. Analyses of the mistakes showed preliminary evidence that priming the participants for certain targets’ body types increased the chance for friendly targets to get shot when their body type partially matched the description, which is in line with priming tests performed in more realistic setups on police officers (Taylor, 2019). The game environment may therefore be suitable to investigate, in future studies, the *in-action* relation between physiology and response inhibition, as well as the effect of biofeedback on performance (Caballero Sánchez et al., 2016). Ideally, associations between in-game behavior and external measures of response inhibition under stress should be demonstrated in a larger sample, with a police relevant transfer condition, to further establish the validity of the in-game behavioral assessments. Importantly, in this game performance was affected by the biofeedback. Indeed, biofeedback was implemented as vision manipulation and thus could impair shooting and target recognition. A visual impairment linked to physiological measures created an artificial link between physiological control and behavioral performance. An illustration of this effect can be shown by fact that the sensitivity *d’* index tended to reduce in sessions with biofeedback. The artificial strengthening of the relation between physiology and behavior is a confound which, together with the changes in game difficulty during a session and the small sample available, made it difficult to investigate links between physiology and performance as have been previously reported (Hashemi et al., 2019). It is, however, anecdotally worth noticing that subject 5, the participant with the highest behavioral performance scores, was also the one with the highest biofeedback score in sessions without biofeedback. This transfer of physiological control skill to sessions where no online feedback on physiology was given suggests that successful transfer of breathing control skill may not come at the cost of reduced performance.

One of the main contributions of the current study was to investigate the proof-of-concept trainability of breathing biofeedback in an active context. Our results suggest that despite the fact that our subjects all received breathing-biofeedback in a classroom setup in the years preceding our experiment, no subject seemed able to control their breathing to satisfying levels prior to the introduction of biofeedback (see Figures 4A, 5). Additionally, our results show support for the trainability in active contexts of deep breathing, thus extending previous evidence showing deep breathing trainability in passive VR contexts (Rockstroh et al., 2019), but also in breaks during real-life police training scenarios (Andersen et al., 2018). Trying to apply emotion regulation techniques *in-action* is not a new idea, and has been implemented with techniques like neurofeedback (Schoneveld et al., 2016), and even breathing biofeedback (Bossenbroek et al., 2020), albeit in a rather meditative VR context. To the best of our knowledge, this is the first attempt at rewarding deep breathing in real-time while immersed in an arousing and active decision-making context. Additionally, only a few participants reached a plateau in biofeedback training after ten sessions. It is therefore unclear when the training benefits of such technique would stop. This issue should be investigated in future research, since there are institutional pressures to shorten training for police officers, sometimes to the point of rendering them useless (Di Nota et al., 2021).

Importantly, although the deep breathing in an active context was shown to reliably increase due to biofeedback, one limitation of this study is that a positive effect of the training on parasympathetic dominance indices could not be demonstrated and should be further investigated in future studies. While no claim can be made regarding parasympathetic control of the heart due to the fact that the heart is controlled by many systems simultaneously (see Hayano and Yuda, 2019, for an overview), we did identify a potentially strong link between adherence to the required breathing pace and a resting function index (the coherence between breathing and low-frequency HRV), as well as with an index of low-frequency HRV. We found no association for high-frequency HRV, which has been linked to increased performance in decision tasks (Gamble et al., 2018) and is usually seen as an index of parasympathetic dominance. The lack of results in high-frequency HRV might have a methodological explanation, as standing has been reported to dampen this HRV metric (Srinivasan et al., 2002). Additionally, training deep breathing transfers the breathing-induced HRV fluctuations from the higher to the lower part of the spectrum, hence increasing low-frequency HRV, possibly at the cost of high-frequency HRV (Hayano and Yuda, 2019). While low-frequency HRV may in fact be driven by parasympathetic nervous system activity during slow breathing (Kromenacker et al., 2018; Schwerdtfeger et al., 2020) it does not allow claims about the influence of the parasympathetic nervous system. Moreover, we found no consistent increase in either low or high frequency HRV across training, only in coherence. While coherence has been linked to improved cognitive and emotional function (Mather and Thayer, 2018; Schwerdtfeger et al., 2020), it is not possible, with the current dataset, to assess if the specific effect we report here is effective in producing such cognitive and emotional improvements.

The present study has other limitations, like the lack of a dedicated breathing pace baseline recording. Mostly, this study is limited due to the small sample size, which prevents not only inquiries on efficiency and training output (Smith and Little, 2018), but also on the interaction between behavior and physiology. A larger and more diverse sample, combined with a non-VR transfer task, would allow us to test the extent to which the training in VR transfers to physiological and behavioral patterns observed in real life, which is a critical test to evaluate the generalizability of the VR measures (Miller et al., 2019). A last limitation is the fact that our sample was, years prior to our training, exposed to a stress reduction course containing psychoeducational material and a short biofeedback session, as part of the mandatory training of all police officers in Netherlands. However, the lack of evidence that the previous training and exposition to psychoeducational theories (that have a high prevalence in police curricula) had any effect in session 1 made us consider it as a non-confounding factor.

In terms of future work, our VR game could also be used to investigate the dynamical interaction between physiological state and decision-making in a more complex and engaging environment than traditional laboratory assessments. Indeed, little is known about the effectiveness of physiological control in the variety of phases through which police action evolves. While a fully realistic VR setup would contain too much variability and dimensions to measure performance reliably (Brehmer, 1992; Michela et al., 2019), testing well-known scientific measurements of decision-making in dynamical interactions could prove invaluable to relate performance and physiological control, but also to further investigate the external validity of those paradigms (Flake et al., 2017). Additionally, we recommend future studies to individualize the biofeedback target to the player, thus rewarding the breathing frequency and associated strategy that more efficiently raises HRV, rather than a fixed target like in the present study. Lastly, the output data includes positional tracking, and could be used to investigate potential movement confounds in the HRV measurement. More information on the ways movement can perturbate HRV biofeedback can be found in our implementation oriented publication (Brammer et al., 2021).

To conclude, while the generalizability of the results presented here needs to be assessed in future studies with larger samples, the present study showed the promise of a VR game to train physiological regulation in an arousing, police-relevant decision-making context. In addition, there seems to be support for targeting the behavioral action mechanisms that were included in the VR game. Our VR design opens new potential avenues for testing the impact of priming, arousal, and physiological control on (police-relevant) behavior in a more naturalistic context than traditional laboratory experiments. It will be important to design future studies to not only assess whether the VR game impacts decision-making under stress for police officers, but also if the physiological mediators of these effects are indeed the same that are targeted in the training.

## Data Availability Statement

The datasets presented in this article are not readily available due to privacy concerns, since the dataset contains performance indexes of police officers. Requests to access the datasets should be directed to the corresponding author, abele.michela@gmail.com.

## Ethics Statement

The studies involving human participants were reviewed and approved by Ethics Committee Social Science, Behavioral Sciences Institute Nijmegen, Thomas van Aquinostraat 4, ecsw@ru.nl. The patients/participants provided their written informed consent to participate in this study.

## Author Contributions

AM, AN, FK, and IG wrote the first draft of the manuscript. AM, JB, JP, MR, WD, KR, FK, and IG conceptualized the VR environment and experiment. WD provided the theoretical policing framework. WD and AS coordinated data collection. JB, JP, and RO conceptualized and implemented the biofeedback. AM, AN, and JB collected the data. AM, AN, JB, MR, and FK analyzed the data. All authors reviewed and contributed to the manuscript. KR, IG, and FK recruited funding for the project.

## Conflict of Interest

The authors declare that the research was conducted in the absence of any commercial or financial relationships that could be construed as a potential conflict of interest.

## Publisher’s Note

All claims expressed in this article are solely those of the authors and do not necessarily represent those of their affiliated organizations, or those of the publisher, the editors and the reviewers. Any product that may be evaluated in this article, or claim that may be made by its manufacturer, is not guaranteed or endorsed by the publisher.

## Footnotes

1 Individual trajectories were plotted alongside the average of all participants, with standard error.
