# Supplementary material for: Deep-Breathing Biofeedback Trainability in a Virtual-Reality Action Game: A Single-Case Design Study With Police Trainers
Source: Front Psychol. 2022 Feb 10;13:806163. doi: 10.3389/fpsyg.2022.806163 (PMC8868154; doi:10.3389/fpsyg.2022.806163)
Supplement: Supplementary file 1 [file Data_Sheet_1.pdf]

Supplementary Material

1 Calculation of the scores presented to the participants at the end of the VR training session.

To give the participants a comprehensive summary of their performance, self-constructed “control scores” were presented at the end of every VR session. Those scores were named to match the way police officers themselves refer to their performance in real life. Namely, they mention that being in control of the self is needed to control the suspect, which is in turn needed to control the situation.

1.1 Control over the situation

Policing theory outlines the need for police officers to keep control of the situation, which entails keeping monitoring their environment and the communication with the commanding center frequent. In our game environment, these two dimensions were translated in 3 metrics, two accounting for environment monitoring (number of times a hostile zombie managed to reach and attack the player and number of times a zombie managed to reach the player unobserved), and 1 for communication (confirming reception of the dispatch information). Each dimension was weighted as 50% of the final “control over the situation” score.
Control over the situation consisted of the number of times a participant was attacked by a zombie, the times a participant was surprised approached (by a zombie which the participant did not see) and the number of radio confirmations (confirming the message of which zombies, based on eye colour, to shoot by pressing a button on the radio). These variables were calculated using the following equations:

- Times attacked by zombie (TA) = (1- ((#first time hit by hostile) / (#total number of hostile)))*100

- Surprise approaches (SA) = (1 – (#hostile AND innocent unseen approaches) / (#total number of zombies))*100

- Radio confirmations (RC) = ((#radio confirmations made) / (#radio confirmations required in the game))*100

- Control over the situation = (TA + SA + 2*RC)/4

1.2 Control over the suspect

This score was summarizing the decision made by the player. The first measure is the arrest fire count. According to the Dutch police theory, police officers are allowed to shoot at the legs of aggressive incoming suspects from la large distance (15m to 25m), while shooting at the chest is recommended at shorter distances (up to 5m, after which other coercion tools are recommended for the officer’s safety). The distance was slightly adapted in the game, but the principle of rewarding long distance shots was kept to encourage anticipating threats. This decision-making aspect was only weakly and linearly rewarded as many occasions to shoot at long distance are provided by the game.

Shooting mistakes were severely punished score-wise. Shooting mistakes entails shooting friendly zombies and shooting zombies before they were assigned an eye color (element allowing the player to decide if a zombie is friendly or not). Those mistakes were punished on a logarithmic scale, which allowed to punish the first mistake rather severely (~20% of final score was lost with the first friendly shooting mistake) and the following ones less severely, so that a player making many mistakes would not end up with a negative score.
Control over the suspect consisted of the amount of arrest fire hits (shooting a zombie from 13-17 meters), too far hits (shooting a zombie when the eye colour is not visible yet), and no-go hits (shooting a zombie with the wrong eye colour (innocent zombie)). These variables were calculated using the following equations:

• Arrest fire hits (AFH) = ((#hostile_hit in the 13-17m range) / (#hostile_hit))* 100

• Too far hits (TFH) = (1 - log10[(#Far_hit +2) / 2])*100

• No-go hits (NGH) = (1 - log10[(#innocent_hit +2) / 2])* 100

• Control over the suspect = (AFH + TFH + 3 * NGH)/5

1.3 Control over the self

Since the point of the training is to enhance awareness on the breathing control during the active decision-making context, this score was made purposefully more difficult to increase, hence nudging the player to pay more attention to this particular aspect of the summary scores for the following sessions. To that end, only moments of high breathing control (scores of 0.8/1 breath control) were counting toward the final "control of the self" score.
Control over the self (i.e. self-regulation of breathing) reflected the proportion of the breathing rate of the participant matching the required breathing pace of four to twelve breaths per minute during that specific period (e.g. wave, session). The biofeedback score was calculated using the following equations:

• Biofeedback score untransformed (BSU) = spectral density over the biofeedback target range of four to twelve breaths per minute / spectral density outside the target range of four to twelve breaths per minute (Brammer et al., 2021).

• Biofeedback score transformed (BST) = (BSU - (min(BSU))) / (max(BSU)) - (min(BSU))

• Control over the self = ((time BST higher than 0.8) / (total time)) * 100

The transformed biofeedback score ranges from 0 to 1. If BSU was larger than 1.5, A BST of 1 was returned since 1 is the maximum biofeedback score.

The process of calculating the biofeedback score is depicted in Figure A.1. Every 2 seconds, a breathing segment of the last 30 seconds of breathing data was analyzed, resulting in an overlap of 28 seconds between consecutive segments. Figures A.1A, A.1B and A.1D show two different breathing

paces: a fast-breathing pace (blue) and a slow breathing pace (orange). Figure A.1B represents a power spectrum in which it is visualized that a breathing rate of 4 to 12 breaths per minute is the rewarded breathing pace. The power spectrum is used to evaluate how much of the current breathing rate matches the biofeedback target of 4 to 12 breaths per minute (the green area) in Figure A.1B. To retrieve the biofeedback score, the green area under the curve (AUC target) is divided by the red area under the curve (AUC outside target). Next, the biofeedback score was standardized, as shown in Figure A.1 (Brammer et al., 2021).

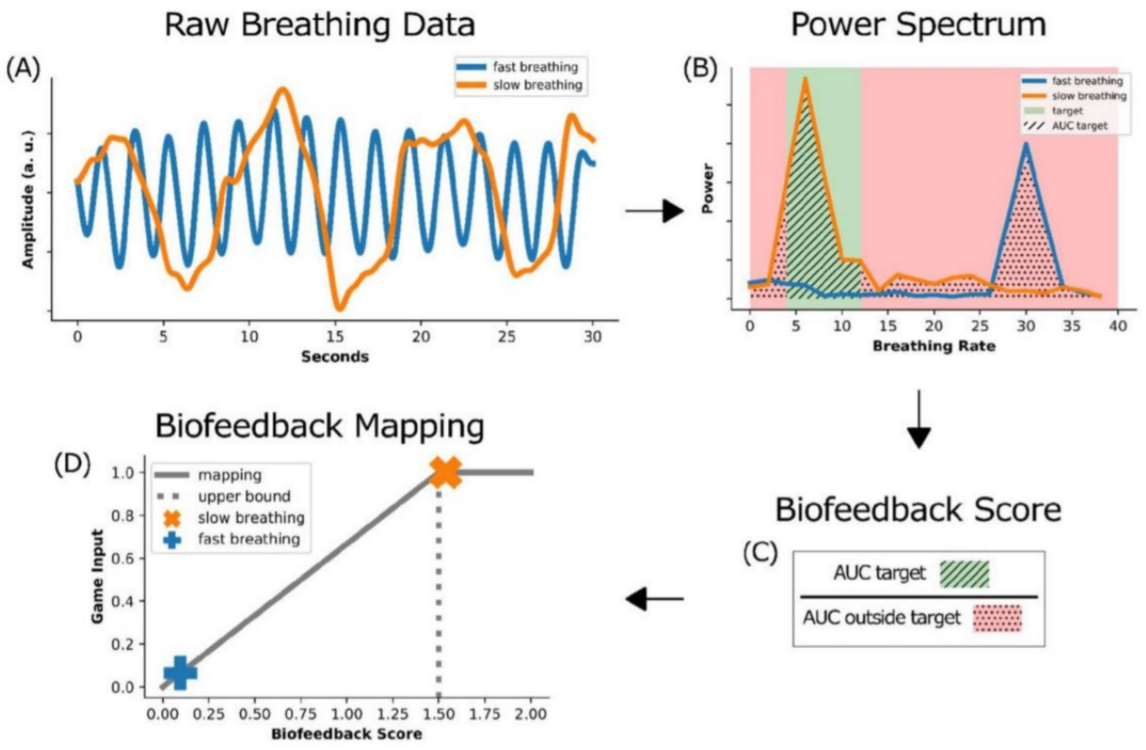

Supplementary Figure 1.1. Reprinted from “Breathing Biofeedback for Police Officers in a Stressful Virtual Environment: Challenges and Opportunities,’’ by J. C. Brammer, J. M. van Peer, A. Michela, M. M. J. W. van Rooij, R. Oostenveld, F. Klumpers, W. Dorrestijn, I. Granic, and K. Roelofs, 2021. Frontiers in Psychology, 12, p. 7 (https://doi.org/10.3389/fpsyg.2021.586553). CC BY, reprinted with permission.

2 Target approach analysis (TAA) and after action report (AAR)

Doel-Aanpak Analyse (TAA) ***(Target approach analysis)***

1. Doel : waar ga je je in deze sessie op richten? (meerdere keuzes zijn mogelijk)
   Goal: what will you focus on in this session? (several choices possible)
   - □ Controle over het zelf *(Control of the self)*
   - □ Controle van de verdachte *(Control of the suspect)*
   - □ Controle van de situatie *(Control of the situation)*

2. Risico's : *(Risks:)*
   Aantal verkeerde doelen ___________________ *(number of wrong targets)*

3. Mag ik: wat is volgens jou de beste score die behaald kan worden in een sessie?
   (Can I: what do you think is the best score that can be achieved in a session?)
   Controle over het zelf _____%
   Controle van de verdachte _____%
   Controle van de situatie _____%
   Aantal verkeerde doelen ____________________

4. Kan ik : wat denk je dat je score zal zijn in deze sessie?
   (Can I: What do you think your score will be in this session?)
   Controle over het zelf _____%
   Controle van de verdachte _____%
   Controle van de situatie _____%
   Aantal verkeerde doelen ____________________

5. Plan van aanpak : *(Action plan:)*
   Wat kan er fout gaan in deze sessie? en hoe ga je erop reageren?
   (What can go wrong in this session? and how are you going to react to it?)

   ____

   ____

   ____

After Action Review (Ging het goed, of ging het niet fout? Goed gegaan of goed gedaan?)
(Did it go right, or didn't it go wrong? Did it go right or did it right?)

1. Heb je veilig gewerkt? *(Did you work safely?)*
   Controle over het zelf _____%
   Controle van de verdachte _____%
   Controle van de situatie _____%
   Aantal verkeerde doelen ____________________
2. Wat maakt dat je wel/niet van je plan bent afgeweken?
   (What makes you deviate/not deviate from the plan?)
   ____
   ____
   ____
3. Wat doe je de volgende weer? *(What will you do again the next time?)*
   ____
   ____
   ____
4. Wat doe je de volgende keer anders? *(What will you do differently next time?)*
   ____
   ____
   ____
5. Wat doe je de volgende keer niet meer? (*What will you not do anymore next time?)*
   ____
   ____
   ____
6. Hoe voel je je? (Ben je klaar voor de volgende ronde?)
   (How do you feel? (Ready fort he next round?))
   ____
   ____

Uit: Beyond the Split Second, W. Dorrestijn 2014-2018. Alle rechten voorbehouden
(From: Beyond the Split Second, W. Dorrestijn 2014-2018. All rights reserved)

3 SCED analysis and tables

Table 3.1

Means and standard deviations of the biofeedback value per phase per participant.

Participant	A_1 phase, mean (SD)	B phases, mean (SD)	$A_{2\text{-}5}$ phases, mean (SD)
1	.002(0.015)	.649(0.247)	.77(0.195)
2	.041(0.122)	.466(0.269)	.391(0.278)
3	.044(0.13)	.497(0.278)	.594(0.266)
4	.129(0.255)	.435(0.274)	.343(0.288)
5	.118(0.209)	.709(0.200)	.725(0.172)
6	.043(0.124)	.356(0.320)	.329(0.309)
7	.193(0.27)	.555(0.287)	.524(0.307)
8	.036(0.135)	.385(0.314)	.265(0.302)
9	.087(0.217)	.424(0.323)	.206(0.28)

Note. A_1 = first phase without biofeedback; B = phases with biofeedback; $A_{2\text{-}5}$ = all phases without biofeedback (the first phase excluded); Biofeedback values can range between 0 and 1.

3.1 Level

To facilitate visual inspection of the evolution of participants' *level* in biofeedback score, figure 4A in the main article displays session averages for each participant. A consistent steep improvement in biofeedback control is clearly visible here between the first and the second training session, where the online biofeedback element is introduced for the first time. All subjects ended the training with a higher breathing score than in the very first sessions, and five subjects (1, 2, 3, 5 and 7) ended the training with a higher breathing score than in the first B-phase, thus suggesting that they learned how to control the biofeedback parameter. Two participants (1 and 5) approached a potential ceiling effect in the second half of the training.

3.2 Trend

The *trends* in biofeedback score within a training session were evaluated by means of a linear regression fitting (MATLAB function "polyfit.m"). A positive trend indicates an improvement in breathing control throughout the training session, whereas a negative trend indicates a decrease in breathing control over time in a session. The differences in trends between subsequent sessions were analyzed (decelerating or accelerating). An accelerating trend reflects a positive training effect since the training is designed to increase the biofeedback values (Lane & Gast, 2014). If the trend of the data changes with a change in condition (either addition or removal of biofeedback), this suggests the training has an effect. When a participant shows three of such changes, the training is deemed significantly effective (Kratochwill et al., 2013).

The *trends* in biofeedback score *within* a training session are presented in Table 6.2. As could be expected since the game gets harder throughout a session, a large proportion of the sessions (63 out

of 90) displayed a negative trend, suggesting that players performed worse in breathing control over time. We also identified when changes in trend direction occurred in consecutive sessions, where biofeedback was added or removed. Five subjects (1, 2, 3, 5 and 7) each showed at least three effects in which the trend changed with a change in condition, thus providing evidence for training effectiveness. No subject except subject 3 displayed a trend in the first session, suggesting a potential floor effect in the first session.

Table 3.2

Slope of the linear regression trend, per session, per participant.

Participant	Session									
	1(A_1)	2(B_1)	3(B_2)	4(A_2)	5(B_3)	6(A_3)	7(B_4)	8(A_4)	9(B_5)	10(A_5)
1	0	.006*	.002	-.002	-.001	-.003*	-.003*	-.001	-.001	0
2	0	.004*	-.002	-.003	-.004*	-.002	0	0	.002	-.001
3	-.001	-.005*	-.002	-.009*	.002	-.002	-.002	-.005*	-.005*	-.003*
4	0	-.004*	-.006*	-.001	-.005*	-.001	-.005*		-.001	-.003
5	0	-.002	-.003	-.004	0	-.003*	.001	0	.001*	0
6	0	-.003	-.004	-.001	-.003	-.005*	-.004	-.007*	-.003	-.002
7	0	-.001	.002	-.001	0	-.004	.001	-.004	-.002	-.005*
8	0	-.007*	-.004	.002	-.006*	-.006*	-.004	-.004	-.006*	-.004
9	0	-.005*	.001	-.002	-.004	-.001	-.001	-.003	-.004*	-.004*

* $p < 0.05$. ** $p < 0.01$. *** $p < 0.001$

Note. A_1 = first phase without biofeedback; B = phases with biofeedback; $A_{2\text{-}5}$ = phases without biofeedback (the first phase excluded); Grey cells indicate the session is part of a change; Data from session 8 of participant 4 is missing due to a technical issue.

3.3 Variability

Variability of the data refers to the fluctuation of the biofeedback scores per session (Horner et al., 2005; Kratochwill et al., 2013). Median absolute deviation (MAD) scores were calculated to compare variability between sessions and A/B phases (Levin et al., 2021). As shown in Table 6.3, the variability of the biofeedback values was higher in sessions with biofeedback (B phases) compared to sessions without biofeedback (A phases) for eight participants (1, 2, 3, 4, 5, 6, 8, and 9). Compared to the first baseline session (A_1), there was more variability in the subsequent sessions, both with (B phases) and without biofeedback ($A_{2\text{-}5}$ phases), in line with the earlier suggestion of a floor effect in the first session.

Table 3.3

Median Absolute Deviation (MAD) per session and phase, per participant

Participant	Session											
	1 (A_1)	2 (B_1)	3 (B_2)	4 (A_2)	5 (B_3)	6 (A_3)	7 (B_4)	8 (A_4)	9 (B_5)	10 (A_5)	$A_{2\text{-}5}$ phases	B phases
1	0	.283	.3	.122	.214	.096	.054	.066	.086	.07	.088	.187
2	0	.244	.243	.067	.245	.062	.222	.224	.146	.149	.125	.22
3	0	.105	.258	.287	.256	.224	.237	.242	.205	.09	.211	.212
4	0	.226	.311	.101	.231	.285	.12		.149	.217	.201	.208
5	0	.219	.192	.3	.143	.164	.082	.047	.018	.01	.13	.131
6	0	.118	.269	.223	.31	.29	.282	.321	.309	.092	.231	.258
7	0	.225	.272	.275	.279	.241	.213	.27	.121	.229	.254	.222
8	0	.249	.276	0	.304	.001	.272	.294	.242	.258	.138	.269
9	0	.274	.135	0	.263	.231	.219	0	.19	.029	.065	.216

Note. A_1 = first phase without biofeedback; B = phases with biofeedback; $A_{2\text{-}5}$ = phases without biofeedback (the first phase excluded). Data from session 8 of participant 4 is missing due to a technical issue.

3.4 Immediacy of effect

The immediacy of the effect of the intervention is usually examined by calculating the difference in level between the last three to five data points of one session and the first three to five data points of the next session to see whether the data immediately changes when biofeedback is either removed or added (Kratochwill et al., 2010). Some drawbacks to this approach are 1) ignoring autocorrelation between observations, 2) not using all the data of a session, and 3) no guidelines on interpreting the magnitude of the difference. Using a Bayesian estimator of abrupt change, seasonality, and trend (termed BEAST-model; Zhao et al., 2019) addresses all those issues. Therefore, the BEAST-model was used to assess the immediacy of effect. The BEAST-model assumes the change point (the change from one session to the next) as unknown and indicates based on the data after which data point in each session there was a substantial change in biofeedback values. The parameters of the BEAST-Model can be found in supplementary material 7. The training is considered effective for a participant when the estimated change point of the model corresponds three times with the actual change point.

The values in Table 6.4 indicate after which data point in each session there was a substantial change in biofeedback values according to the BEAST-model. Changes detected between session 2 and 3 were discarded, as both sessions are B phases. Eight participants (1, 2, 3, 4, 5, 6, 7 and 8) each showed at least three instances at which the estimated change point occurred at the true moment of change from one session to another (up to 10 samples before the end, or 3 samples after the start of a session).

Table 3.4

Estimations of the Bayesian change-points model transitions of A-to-B and B-to-A phases.

Subject	Sample of detected change in phase (distance from real sample)							
	1	2	3	4	5	6	7	8
1	82(10)	147(3)	210(-6)	353(-7)	422(62)	506(2)	619(43)	
2	76(4)	148(4)	290(2)	351(-9)	426(-6)	506(2)	570(-6)	650(2)
3	64(-8)	199(55)	338(50)	426(-6)	507(3)	660(12)		
4	74(2)	139(67)	208(64)	334(46)	434(2)	502(-2)	615(39)	
5	74(2)	136(64)	249(33)	321(33)	384(24)	497(-7)	650(2)	
6	106(34)	224(8)	290(2)	353(-7)	418(58)	506(2)	567(-9)	633(57)
7	73(1)	150(6)	213(-3)	281(-7)	362(2)	491(59)	611(35)	
8	72(0)	193(49)	290(2)	353(-7)	434(2)	536(32)	599(23)	660(12)
9	138(66)	202(58)	290(2)	377(17)	474(42)	536(32)	602(26)	

Note. A seasonal transition is considered to be correctly detected if placed up to 10 samples before the end, or 3 samples after start of a phase. The seasons to be detected are 72 samples long, each sample represents 15 seconds of gameplay; Grey cells indicate a correctly detected phase change

3.5 Summary of visual analysis

To summarize the visual analysis, all participants except subject 4 showed at least three effects for at least one index of the visual analysis. Four participants (1, 2, 5 and 7) reached formal significance as they showed effects in at least three main aspects of the visual analysis: trend, immediacy of effect and non-overlap of data. This result corresponds to a moderate positive evidence of an intervention effect, according to the guidelines for SCED analysis (Kratochwill et al., 2013). An overview, per participant, of how many replications of an effect for each main aspect of the visual analysis is included in Table 6.5.

Table 3.5
Overview of the replications of an effect for each main aspect of the visual analysis

Participant	Level	Variability	Trend	Non-overlap	Immediacy of effect	Positive effects	
						triple repetitions	overall
1	Y	Y	5	3	3	3	5
2	Y	Y	6	5	6	3	5
3	Y	N	4	2	3	2	3
4	N	Y	1	2	3	1	2
5	Y	N	8	5	3	3	4
6	N	Y	1	4	4	2	3
7	Y	N	7	3	4	3	4
8	N	Y	3	7	4	2	3
9	N	Y	3	5	1	1	2

Note. Grey cells indicate the presence of an effect for the aspects requiring a triple effect replication; Y = a positive effect was present; N a positive effect was not present

4 Threat and HRV analyses

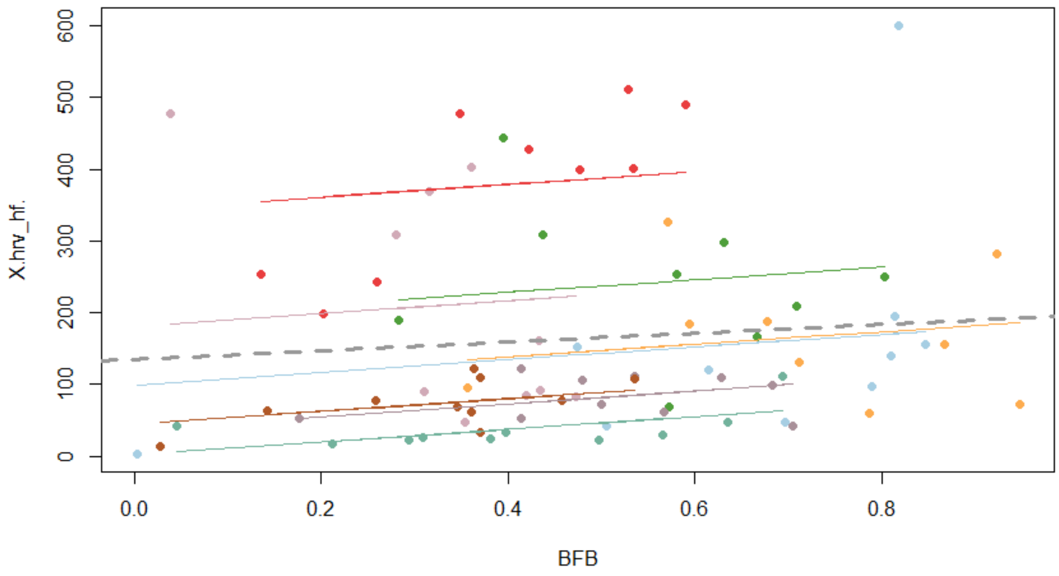

Supplementary Figure 4.1. Repeated measures correlation between High-frequency HRV and biofeedback score, evaluated at the session level; X.hrv_hf = high-frequency HRV; BFB = biofeedback score.

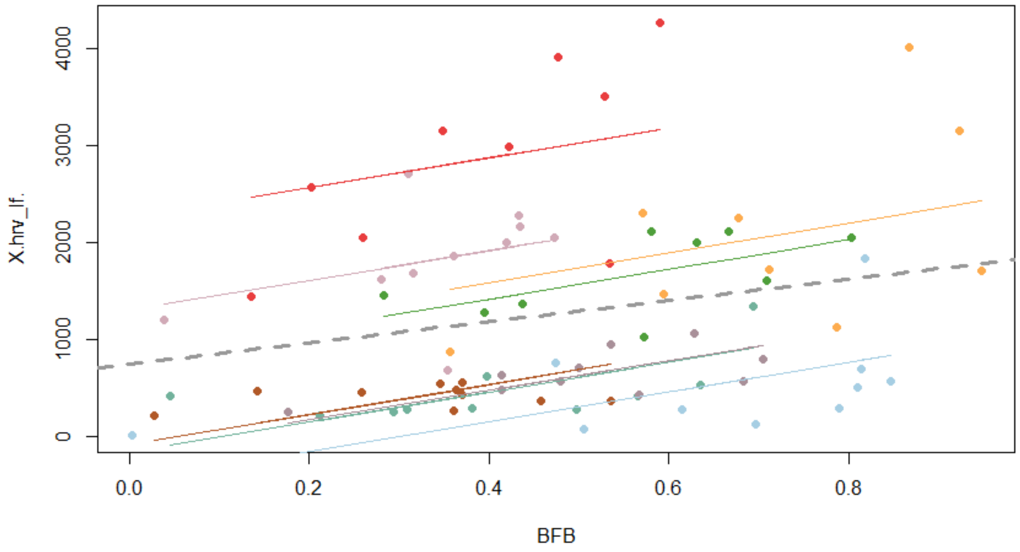

Supplementary Figure 4.2. Repeated measures correlation between Low-frequency HRV and biofeedback score, evaluated at the session level; X.hrv_lf = low-frequency HRV; BFB = biofeedback score.

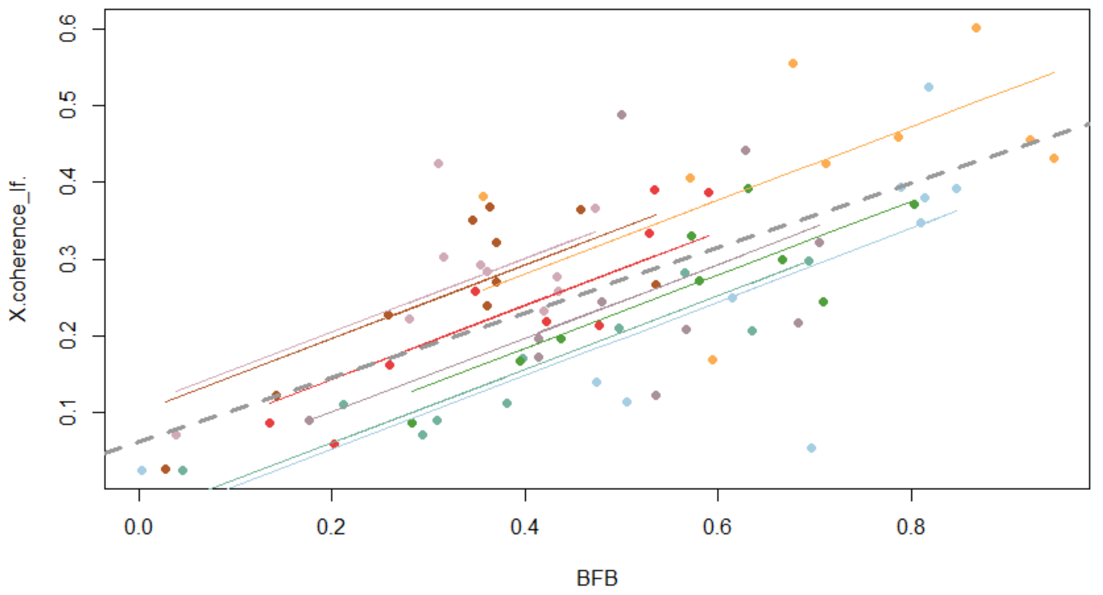

Supplementary Figure 4.3. Repeated measures correlation between the low-frequency HRVcoherence with breathing and biofeedback score, evaluated at the session level; X.coherence_lf = low-frequency HRV coherence with breathing pace; BFB = biofeedback score.

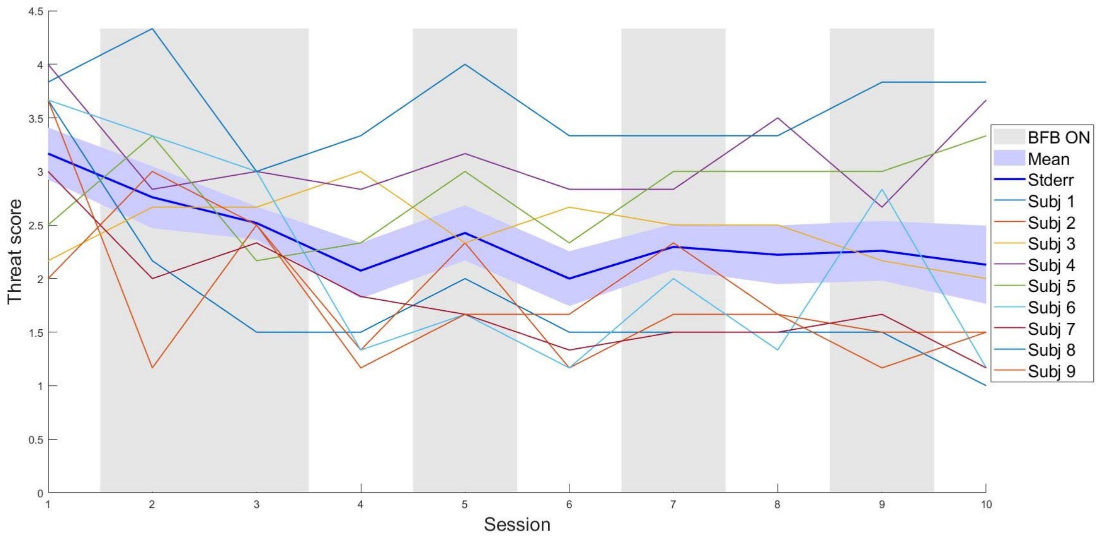

Supplementary Figure 4.4. Evolution of the threat-subscale of the Threat-Challenge appraisal questionnaire; Stderr = standard error of the mean; BFB ON = Sessions in which online biofeedback was presented to the participants.

5 Target approach analysis (TAA) and after-action review (AAR) results

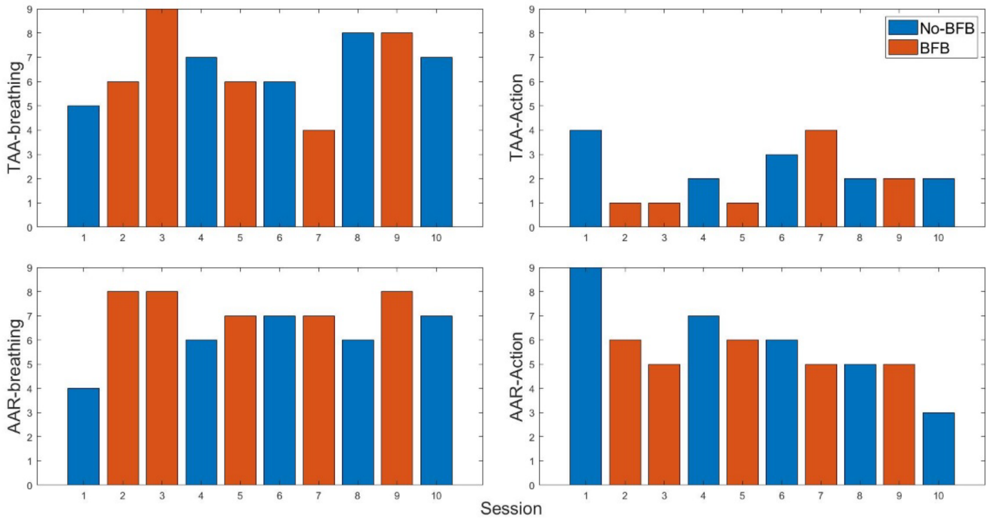

Supplementary Figure 5.1. The general occurrence of breathing and action-related keywords in the TAA and AAR questionnaires; No-BFB = Sessions without online biofeedback; BFB = Sessions with online biofeedback; -breathing = occurrences of participants mentioning breathing or biofeedback related keywords; -Action = occurrences of participants mentioning action related keywords.

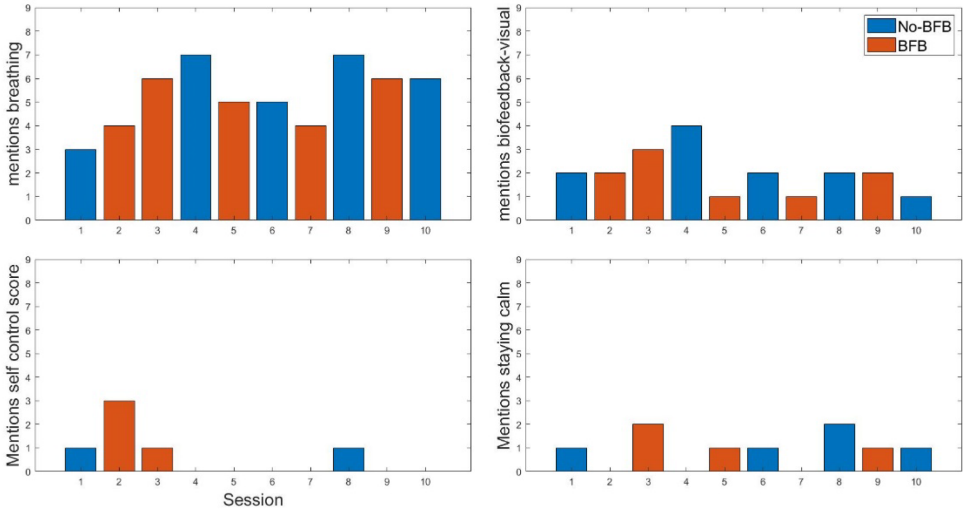

Supplementary Figure 5.2. The detailed occurrence of breathing-related keywords in the TAA questionnaire; No-BFB = Sessions without online biofeedback; BFB = Sessions with online biofeedback; mentions breathing = occurrences of participants mentioning breathing or respiration related keywords; mentions biofeedback-visual = occurrences of participants mentioning the graphical implementation of online biofeedback as visual impairment; mentions self-control score = occurrences of participants mentioning the self-control score

directly; mentions staying calm = occurrences of participants mentioning the self-control score indirectly by referring at the state of calmness.

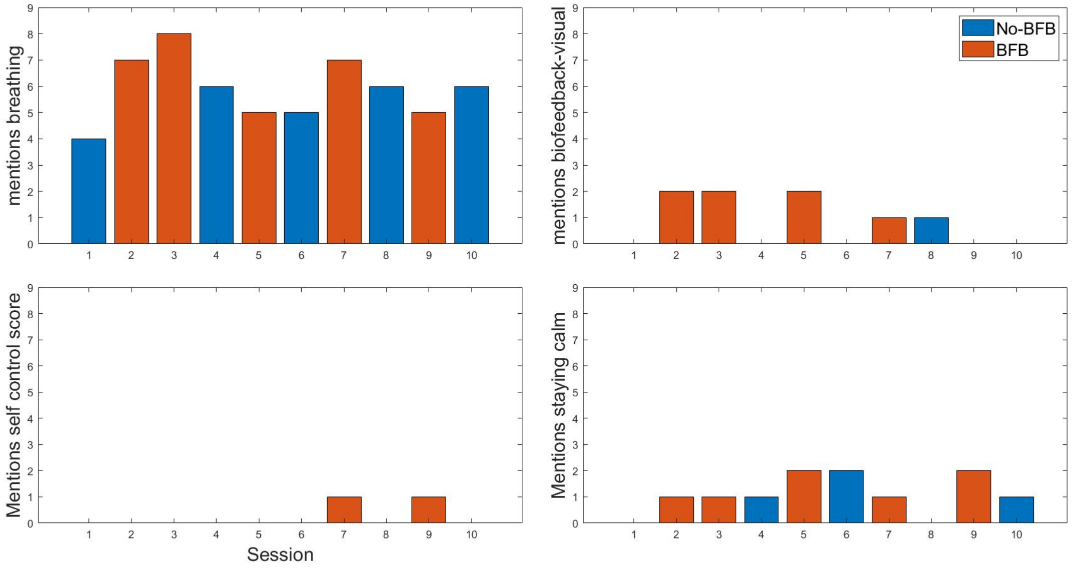

Supplementary Figure 5.3. The detailed occurrence of breathing-related keywords in the AAR questionnaire; No-BFB = Sessions without online biofeedback; BFB = Sessions with online biofeedback; mentions breathing = occurrences of participants mentioning breathing or respiration related keywords; mentions biofeedback-visual = occurrences of participants mentioning the graphical implementation of online biofeedback as visual impairment; mentions self-control score = occurrences of participants mentioning the self-control score directly; mentions staying calm = occurrences of participants mentioning the self-control score indirectly by referring at the state of calmness.

6 behavioral priming effects

Table 6.1

Go/nogo actions distributions across sessions and subjects. Columns are sub-divided according to the large identifier of the zombie related to the trial.

	Hit	CR	FA	Miss	Total
large ID	18169	5234	252	10708	34363
other ID	693	4718	122	576	6109
Total	18862	9952	374	11284	40472

Note. Hit = hit or true positives (hostile zombie shot before reaching player); CR = correct rejection or true negative (innocent zombie reaching player unharmed); FA = false alarm (innocent zombie shot before reaching player); Miss = miss or false negative (hostile zombie reaching player unharmed); large ID = has a large identifier (body type) announced as presumably hostile in radio dispatch; other ID = does not have a large identifier (body type) announced as presumably hostile in radio dispatch.

7 BEAST-model parameters

```
%% Set up the parameters needed for the BEAST algorithm
% Some of these parameters are the model specficiation parameters of BEAST
% (e.g., minSeasonOrder, maxSeasonOrder, minSetpDist_trend,
% minSepDist_Season); other parameters are just some input variables to
% control simulation behaviors or program outputs (e.g., samples,
% thinningFactor, seed, computeCredible).
%
opt.period    = 72;
opt.minSeasonOrder = 0;
opt.maxSeasonOrder = 1;
opt.minTrendOrder=0;
opt.maxTrendOrder=1;
opt.minSepDist_Trend  =  30;
opt.minSepDist_Season =  60;
opt.maxKnotNum_Trend =  10;
opt.maxKnotNum_Season = 10;
opt.maxMoveStepSize   = 10;
opt.samples = 720;
opt.thinningFactor = 1;
opt.burnin = 200;
opt.chainNumber=2;
opt.resamplingTrendOrderProb=0.2;
opt.resamplingSeasonOrderProb=0.17;
opt.omissionValue=-999;
opt.seed=100;
opt.computeCredible=0;
opt.computeSlopeSign=1;
opt.algorithm='beast';
opt.computeHarmonicOrder=1;
opt.computeTrendOrder=1;
opt.computeChangepoints=1;
%opt.timeDimensionIndex=3;
%% Run BEAST on "Y"
out=beast_default(Y, opt);
% extract "seasonal changes"
scp(f,:)=sort(out.scp);
% check if they fall in the 72 sample +3-10 range -> transition detected
```

8 References

Brammer, J. C., van Peer, J. M., Michela, A., van Rooij, M. M. J. W., Oostenveld, R., Klumpers, F., Dorrestijn, W., Granic, I., & Roelofs, K. (2021). Breathing Biofeedback for Police Officers in a Stressful Virtual Environment: Challenges and Opportunities. *Frontiers in Psychology*, *12*, 586553. https://doi.org/10.3389/fpsyg.2021.586553

Horner, R. H., Carr, E. G., Halle, J., Mcgee, G., Odom, S., & Wolery, M. (2005). The use of single-subject research to identify evidence-based practice in special education. In *Exceptional Children* (Vol. 71, Issue 2, pp. 165–179). SAGE PublicationsSage CA: Los Angeles, CA. https://doi.org/10.1177/001440290507100203

Kratochwill, T. R., Hitchcock, J. H., Horner, R. H., Levin, J. R., Odom, S. L., Rindskopf, D. M., & Shadish, W. R. (2013). Single-Case Intervention Research Design Standards. *Remedial and Special Education*, *34*(1), 26–38. https://doi.org/10.1177/0741932512452794

Lane, J. D., & Gast, D. L. (2014). Visual analysis in single case experimental design studies: Brief review and guidelines. In *Neuropsychological Rehabilitation* (Vol. 24, Issues 3–4, pp. 445–463). Neuropsychol Rehabil. https://doi.org/10.1080/09602011.2013.815636

Levin, J. R., Ferron, J. M., & Gafurov, B. S. (2021). Investigation of Single-Case Multiple-Baseline Randomization Tests of Trend and Variability. *Educational Psychology Review*, *33*(2), 713–737. https://doi.org/10.1007/s10648-020-09549-7

Wetten.nl. (n.d.). *Regeling - Ambtsinstructie voor de politie, de Koninklijke marechaussee en andere opsporingsambtenaren - BWBR0006589*. Retrieved December 20, 2021, from https://wetten.overheid.nl/BWBR0006589/2021-10-01

Zhao, K., Wulder, M. A., Hu, T., Bright, R., Wu, Q., Qin, H., Li, Y., Toman, E., Mallick, B., Zhang, X., & Brown, M. (2019). Detecting change-point, trend, and seasonality in satellite time series data to track abrupt changes and nonlinear dynamics: A Bayesian ensemble algorithm. *Remote Sensing of Environment*, *232*, 111181.
